# Tau isoform-specific enhancement of L-type calcium current and augmentation of afterhyperpolarization in rat hippocampal neurons

**DOI:** 10.1038/s41598-022-18648-0

**Published:** 2022-09-08

**Authors:** Georgiana F. Stan, Timothy W. Church, Ellie Randall, Jenna R. M. Harvey, Jon T. Brown, Kevin A. Wilkinson, Jonathan G. Hanley, Neil V. Marrion

**Affiliations:** 1 School of Physiology, Pharmacology and Neuroscience, University of Bristol, Bristol, BS8 1TD UK; 2 School of Biochemistry, University of Bristol, Bristol, BS8 1TD UK; 3 University of Exeter Medical School, Hatherly Building, Exeter, EX4 4QJ UK; 4 Present Address: Neuroscience, Physiology and Pharmacology, Division of Biosciences, Faculty of Life Sciences, University College London, Gower Street, London, WC1E 6BT UK; 5 Present Address: Cerevance Ltd., 418 Cambridge Science Park, Milton Road, Cambridge, CB4 0PZ UK

**Keywords:** Cellular neuroscience, Ion channels in the nervous system, Molecular neuroscience

## Abstract

Accumulation of tau is observed in dementia, with human tau displaying 6 isoforms grouped by whether they display either 3 or 4 C-terminal repeat domains (3R or 4R) and exhibit no (0N), one (1N) or two (2N) N terminal repeats. Overexpression of 4R0N-tau in rat hippocampal slices enhanced the L-type calcium (Ca^2+^) current-dependent components of the medium and slow afterhyperpolarizations (AHPs). Overexpression of both 4R0N-tau and 4R2N-tau augmented Ca_V_1.2-mediated L-type currents when expressed in tsA-201 cells, an effect not observed with the third 4R isoform, 4R1N-tau. Current enhancement was only observed when the pore-forming subunit was co-expressed with Ca_V_β3 and not Ca_V_β2a subunits. Non-stationary noise analysis indicated that enhanced Ca^2+^ channel current arose from a larger number of functional channels. 4R0N-tau and Ca_V_β3 were found to be physically associated by co-immunoprecipitation. In contrast, the 4R1N-tau isoform that did not augment expressed macroscopic L-type Ca^2+^ current exhibited greatly reduced binding to Ca_V_β3. These data suggest that physical association between tau and the Ca_V_β3 subunit stabilises functional L-type channels in the membrane, increasing channel number and Ca^2+^ influx. Enhancing the Ca^2+^-dependent component of AHPs would produce cognitive impairment that underlie those seen in the early phases of tauopathies.

## Introduction

Neuronal excitability is regulated by the generation of afterhyperpolarizations (AHPs) that hyperpolarize the membrane away from the threshold of action potential initiation and inhibit firing. Hippocampal pyramidal neurons possess 3 phases of AHP, designated fast, medium and slow. The fast AHP regulates action potential firing at the onset of a burst^1^, while the medium and slow AHPs regulate action potential firing over 100 s of msec and seconds respectively^2,3^. The medium AHP is proposed to be produced by activation of Ca^2+^-dependent, apamin-sensitive SK channels and M-current, and deactivation of H-current^3,4^. In contrast, the identity of the channel underlying the slow AHP has proved elusive, with activation of M-channels (Kv7)^5^, the Na^+^-K^+^ ATPase pump^6^ or IK_Ca_ channels^7^ being proposed. Despite any uncertainty of the identity of the Ca^2+^-dependent channel, it is accepted that it is Ca^2+^ entry only through L-type channels that is required to generate both the Ca^2+^-dependent component of the medium AHP and the slow AHP^8–13^.

The amplitude of the slow AHP increases with age^10,14,15^, which can influence learning^16^. Learning of a simple associative learning task is impeded in aged rabbits that exhibit a large slow AHP^10,14,15^. Inhibition of L-type Ca^2+^ channels by nimodipine reduced the amplitude of the slow AHP^8^ and enabled aged animals to learn as well as young animals^14,17^. The increase in the amplitude of the nimodipine-sensitive slow AHP in aged animals likely results from an increase in the number of functional L-type Ca^2+^ channels^18,19^.

It has been common to study transgenic animals as models of tauopathies^20^. However, most transgenic models cannot faithfully mimic sporadic diseases. Sporadic Alzheimer’s disease (AD) is characterized by the presence of neurofibrillary tangles of hyperphosphorylated tau^21^. There are six isoforms of tau in the human CNS. Isoforms are named after the number of near-N-terminal inserts (0N, 1N, 2N) and C-terminal repeats (3R and 4R)^22^. The balance between 3 and 4R isoforms of tau can change, with the ratio of 4R to 3R isoforms increased in the hippocampus and midfrontal cortex of AD patients^23^. Increasing expression of 4R relative to 3R isoforms of tau caused severe seizures and nesting behaviour abnormalities in mice^24^, while overexpression of human 2N4R-tau produced synaptic dysfunction and memory deficits^25^. However, the effect of acute expression of 4R-tau isoforms on neuronal excitability has yet to be determined. Here, we report that overexpression of the most abundant isoform in aged human brain, 4R0N-tau^26^, augments AHPs following a train of action potentials. This effect was specific on both AHP components that are dependent on Ca^2+^ entry through L-type channels. Expression of 4R0N- or 4R2N-tau, but not 4R1N-tau, augmented Ca_V_1.2-mediated L-type channel current in a Ca_V_β-specific manner. Enhancement of Ca_V_1.2-mediated current resulted from an increase in the number of functional channels, and only occurred when the L-type channel was comprised of Ca_V_β3 and not Ca_V_β2a subunits. Co-immunoprecipitation of 4R0N-tau and Ca_V_β3 suggests that the two proteins might be in direct contact. These data demonstrate that accumulation of 4R-tau isoforms in hippocampal neurons increases the number of functional L-type Ca^2+^ channels in the membrane, increasing Ca^2+^ influx. This increased influx of Ca^2+^ enhances the Ca^2+^-dependent components of AHPs that would reduce action potential firing and lead to cognitive decline^25^.

## Results

### Expression of 4R0N-tau augments AHPs in hippocampal neurons

The effect of 4R0N-tau expression on the medium and slow AHPs was examined in CA1 pyramidal neurons in organotypic hippocampal slices 24 h after slices were transduced with control EGFP-alone or EGFP + 4R0N-tau. The amplitude of both AHPs and the membrane currents underlying them were compared between control cells that over-expressed EGFP alone, and 4R0N-tau cells that expressed both the tau isoform and the marker EGFP.

**Figure 1:**
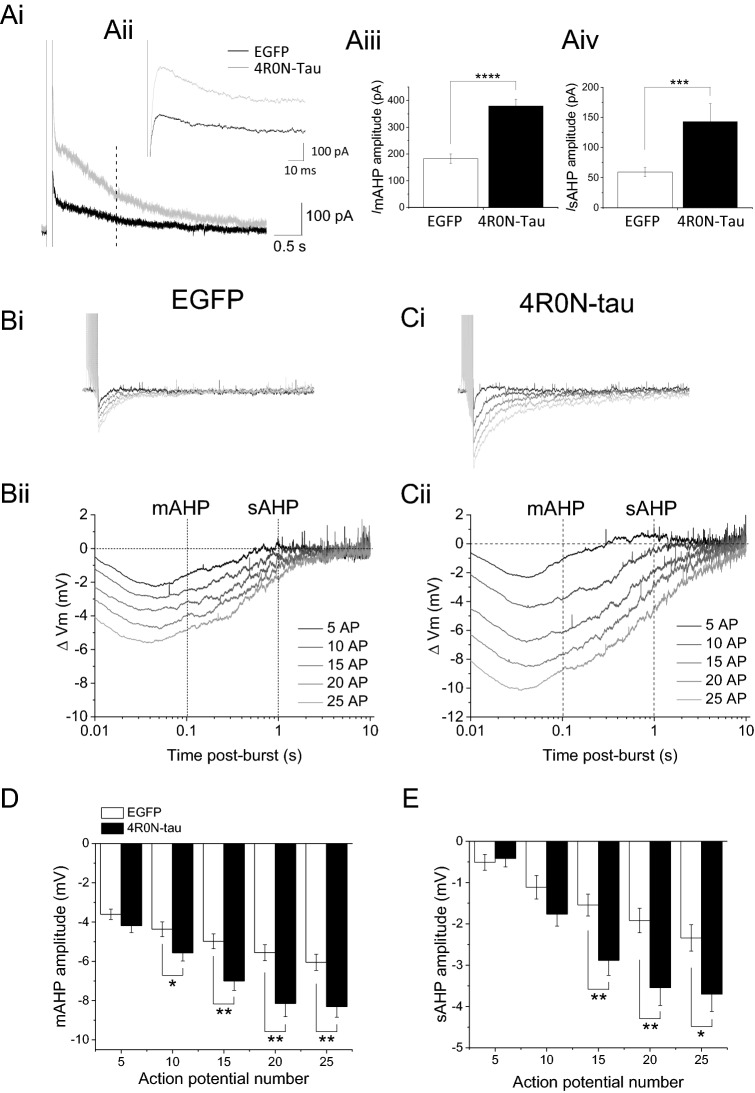
Expression of 4R0N-tau augments the afterhyperpolarization in hippocampal CA1 pyramidal neurons. (**Ai**) Membrane current evoked by a step depolarization to + 10 mV (100 ms duration) and measured at the holding potential of − 50 mV. Current represents that underlying the medium and slow AHPs, with the current underlying the medium AHP shown at higher resolution in (**Aii**). Expression of 4R0N-tau produced a larger amplitude of current underlying both components of AHP (gray trace), when compared with current evoked in cells expressing only EGFP (black trace). (**Aiii**) Bar chart showing augmentation of the current underlying the medium AHP by expression of 4R0N-tau (mean ± s.e.m) (*p* < 0.0001). (**Aiv**) Expression of 4R0N-tau significantly augmented the amplitude of the current underlying the slow AHP (*p* = 0.00097) (EGFP n = 53, 4R0N-tau n = 33). (**Bi,Ci**) Membrane voltage traces showing AHP amplitude increases with action potential (AP) number (5–25 action potentials) in both EGFP-expressing (**Bi**) and 4R0N-tau-expressing (**Ci**) CA1 pyramidal neurons. (**Bii**,**Cii**) Voltage traces from (**Bi**,**Ci**) plotted on a log_10_ time scale for greater clarity of the medium AHP and slow AHP profiles for a cell expressing EGFP alone (**Bii**) and for a cell expressing 4R0N-tau (**Cii**). The medium AHP is measured 100 ms after the last action potential, while the slow AHP was measured 1 s after the last AP. The increase in the medium (**D**) and slow (**E**) AHP components with increasing AP number is augmented in cells expressing 4R0N-tau, when compared with cells expressing EGFP alone. Two-way RM ANOVA with post-hoc LSD *t*-test. EGFP n = 58; 4R0N-tau n = 36; *p ≤ 0.05, **p ≤ 0.01.

The currents underlying the medium and slow AHPs can be resolved under voltage-clamp following a depolarising prepulse from a negative holding potential (− 50 mV). Evoked current was augmented in cells expressing human 4R0N-tau compared with cells expressing EGFP alone (termed control) (Fig. 1Ai). Measurement of the current underlying the medium AHP (I_mAHP_) (Fig. 1Aii) showed it was augmented by 108% in cells expressing 4R0N-tau (Fig. 1Aiii) (EGFP control 182.3 ± 17.8 pA, n = 53, 4R0N-tau-expressing 376.1 ± 25.1 pA, n = 33, *p* < 0.0001, unpaired two-tailed Student’s *t*-test). The current underlying the slow AHP (I_sAHP_) was augmented by 104.8% in 4R0N-tau-expressing cells compared with control (Fig. 1Aiv) (EGFP control 59.3 ± 7.6 pA, n = 53, 4R0N-tau 143.3 ± 28.7 pA, n = 33, *p* < 0.0001, unpaired two-tailed Student’s *t*-test). As mentioned previously, I_mAHP_ is comprised of voltage-dependent M- and H-currents, and voltage-independent SK current^3,4^. Macroscopic current evoked by depolarizing voltage steps from − 80 to + 40 mV from a holding potential of − 80 mV, was not different between cells expressing human 4R0N-tau and EGFP and those expressing EGFP alone (Fig. S2, *F*_(12, 86)_ = 0.32; *p* = 0.98; two-way repeated measures ANOVA with Bonferroni corrections; EGFP n = 59, 4R0N-tau n = 29). These data suggest that augmentation of I_mAHP_ by expression of human 4R0N is not mediated by changes in amplitude of voltage-dependent currents.

**Figure 2:**
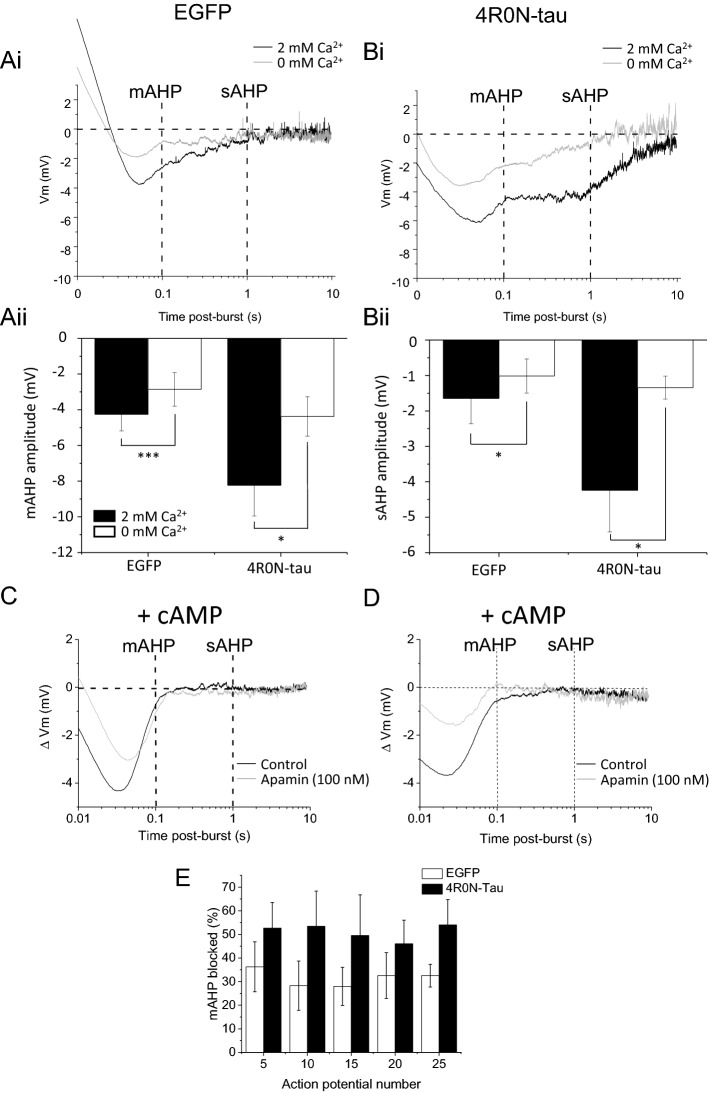
Ca^2+^-dependent components of hippocampal CA1 pyramidal cell AHPs are enhanced by expression of 4R0N-tau. Removal of extracellular Ca^2+^ reduced the amplitude of the medium AHP and abolished the slow AHP in both neurons expressing EGFP alone (**Ai**) and 4R0N-tau (**Bi**). Black traces are control and the gray traces are in the absence of extracellular Ca^2+^. The reduction in amplitude of both the medium AHP (**Aii**) and the slow AHP (**Bii**) is greater in those CA1 neurons expressing 4R0N-tau compared with expressing EGFP alone. (**C**,**D**) Membrane voltage traces of AHP evoked following a train of 15 action potentials (evoked by 2 nA, 2 ms duration current injections delivered at 50 Hz) displayed on a log_10_ time base from cells dialysed with cAMP (1 mM) to abolish the slow AHP in neurons expressing EGFP alone (**C**) or 4R0N-tau (**D**). Application of apamin (100 nM) to inhibit the SK channel-dependent component of the medium AHP produced a larger suppression of the medium AHP (gray traces) in neurons expressing 4R0N-tau (**D**) compared with EGFP alone (**C**). (**E**) The amount of the medium AHP that was blocked by apamin was independent of the number of action potentials used to evoke the slow afterpotential. Plotted are means ± s.e.m. *p* ≤ 0.05, **p ≤ 0.01.

The effect of tau expression can be observed in current clamp recordings, with the firing of a train of action potentials generating an AHP that is comprised of two components, the medium and slow AHPs (Fig. 1B). Expression of 4R0N-tau caused both AHP components to be enhanced following a train of 15 action potentials, with the medium AHP increasing in amplitude by 40.5% (control − 5.0 ± 0.4 mV, n = 58, 4R0N-tau − 7.0 ± 0.5 mV, n = 36, *p* = 0.001, Bonferroni *t*-test), and the slow AHP being enhanced by 86.6% (EGFP control − 1.5 ± 0.3 mV, n = 58, 4R0N-tau − 2.9 ± 0.4 mV, n = 36, *p* = 0.003, Bonferroni *t*-test) (Fig. 1Bi,ii,Ci,ii). The amplitude of both the medium and slow AHPs was dependent on the number of action potentials in the activating train, and enhanced amplitudes of both components were observed with each activating train (Fig. 1D,E) (medium AHP F_(1, 92)_ = 9.1, *P* = 0.003, two-way repeated measures ANOVA with Bonferroni corrections; slow AHP F _(1, 92)_ = 5.4, *p* = 0.02; two-way repeated measures ANOVA with Bonferroni corrections). The 4R0N-tau-enhanced amplitudes of both the medium and slow AHPs result from augmentation of the Ca^2+^-dependent components (Fig. 2). Removing external Ca^2+^ reduced the medium AHP in control cells by 39.6 ± 11.0% (− 4.3 ± 0.9 vs − 2.9 ± 0.9 mV, n = 6, *p* = 0.0008) (Fig. 2Ai,Aii) and the slow AHP by 54.8 ± 13.2% (− 1.6 ± 0.7 vs − 1.0 ± 0.5 mV, n = 6, *p* = 0.045) (Fig. 2Ai,Bii) evoked by a train of 15 action potentials. In 4R0N-tau expressing cells, Ca^2+^-free external solution reduced the medium AHP by 46.2 ± 6.6% (Fig. 2Bi,Aii) (− 8.2 ± 1.7 vs − 4.4 ± 1.1 mV, n = 5, *p* = 0.03) and the slow AHP by 64.9 ± 6.7% (− 4.2 ± 1.2 vs − 1.3 ± 0.3 mV, n = 5, *p* = 0.037,) (Fig. 2Bi,Bii). Importantly, the amplitude of the medium and slow AHP that remained in the absence of extracellular Ca^2+^ was not significantly different between control and 4R0N-tau expressing cells (medium AHP *P* = 0.35, slow AHP *p* = 0.6, unpaired two-tailed Student’s *t*-test). These data indicate that the enhanced amplitudes of both the medium and slow AHPs seen in 4R0N-tau expressing neurons result from an augmented Ca^2+^-dependent component (Fig. 2A,B).

Finally, we wished to confirm that overexpression of human 4R0N-tau enhances the Ca^2+^-dependent SK channel-mediated component of the medium AHP. Our previous data demonstrated that the SK channel component of the medium AHP was comprised of homomeric SK2 channels, which were blocked by apamin with an IC_50_ of approximately 55 pM^27^. The medium AHP was isolated by dialyzing neurons with an electrode solution supplemented with cAMP (1 mM) to block the slow AHP^27^. Application of apamin (100 nM) reduced the amplitude of the medium AHP by 28.0 ± 8.0% in control cells (− 4.4 ± 0.5 vs − 3.1 ± 0.4, n = 3. AHP evoked by 15 action potentials) (Fig. 2C,E). The addition of apamin (100 nM) to 4R0N-tau neurons produced a greater inhibition of the afterpotential (49.5 ± 17.2%; − 4.9 ± 1.0 vs − 2.1 ± 0.6, n = 5. AHP evoked by 15 action potentials; *p* = 0.049) than observed with control cells (Fig. 2D,E). A similar enhancement of block by apamin was observed for AHPs evoked by each number of action potentials in 4R0N neurons (Fig. 2E). This increased inhibition by apamin confirms that overexpression of 4R0N-tau augmented the Ca^2+^-dependent SK channel-mediated component of the medium AHP.

### The 4R0N-tau-evoked enhanced Ca^2+^-dependent component of AHPs is mediated by L-type Ca^2+^ channels

**Figure 3:**
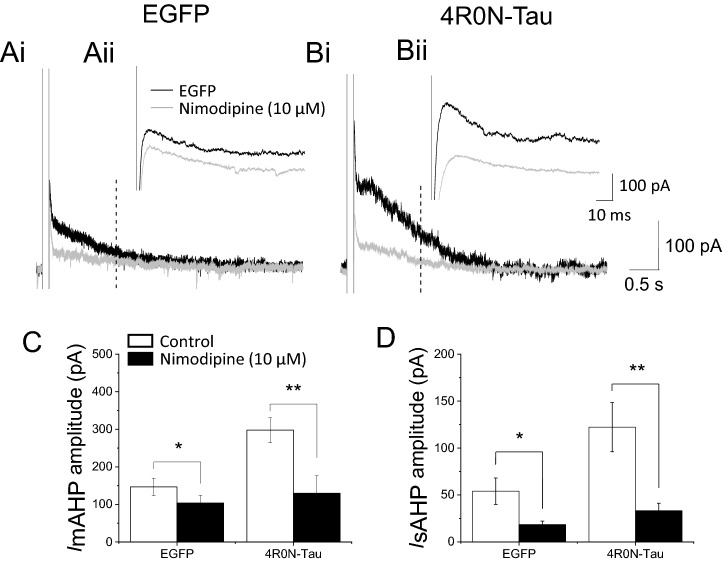
Enhanced Ca^2+^ entry through L-type channels results in augmentation of currents underlying medium and slow AHPs in hippocampal CA1 pyramidal neurons expressing 4R0N-tau. (**Ai,Bi**) Membrane current evoked following a depolarizing voltage step to + 10 mV (100 ms duration) in absence (black trace) and presence (gray trace) of nimodipine (10 μM) in cells expressing either EGFP only (**Ai**) or 4R0N-tau (**Bi**). (**Aii,Bii**) show in higher resolution the current underlying activation of the medium AHP in the absence and presence of the L-type channel inhibitor in cells expressing EGFP (**Aii**) or 4R0N-tau (**Bii**). (**C**) Application of nimodipine (10 μM) reduced the amplitude of the current underlying the medium AHP in both EGFP-expressing (n = 4, *p* = 0.048) and 4R0N-tau-expressing (n = 6, *p* = 0.001) cells. (**D**) Nimodipine (10 μM) significantly reduced the amplitude of the current underlying the slow AHP in both EGFP-expressing (n = 4, *p* = 0.043) and 4R0N-tau-expressing (n = 6, *p* = 0.007) neurons. The effect of nimodipine was greater in 4R0N-tau-expressing neurons. *p* ≤ 0.05, ** *p* ≤ 0.01.

The Ca^2+^-activated potassium channels underlying the two components of AHP in CA1 hippocampal pyramidal neurons are known to be different, with activation of SK channels contributing to the medium AHP^27^ and a channel of unknown identity underlying the slow AHP [but see 7]. It is unlikely that two different types of Ca^2+^activated channel would be affected directly by expression of tau; instead, it is more likely that augmentation of the Ca^2+^-dependent components of AHPs results from an increase in Ca^2+^ entry. The Ca^2+^-dependent component of the medium AHP and the slow AHP is suppressed by the dihydropyridine nimodipine^8,10,11^. The observation that the Ca^2+^-dependent potassium currents underlying the two AHP components are insensitive to blockers of other Ca^2+^ channel subtypes indicates that they are solely dependent on Ca^2+^ entry through L-type channels^11^. Therefore, it is possible that overexpression of 4R0N-tau enhances L-type Ca^2+^ current to produce augmentation of both the Ca^2+^-dependent component of the medium AHP and the entire slow AHP. Both I_mAHP_ and I_sAHP_ were sensitive to nimodipine in control cells (n = 5), with 10 μM nimodipine reducing I_mAHP_ by 29.7 ± 6.1% (I_mAHP_ 164.5 ± 23.4 vs 115.5 ± 17.7 pA; *p* = 0.016, paired two-tailed Student’s *t*-test) and I_sAHP_ by 64.3 ± 5.0%, (73.0 ± 20.2 vs 23.0 ± 5.2 pA; *p* = 0.04 , paired two-tailed Student’s *t*-test). (Fig. 3A,C,D). Nimodipine reduced the I_mAHP_ by a greater magnitude in neurons that overexpressed 4R0N-tau, compared with control neurons. Expression of 4R0N-tau augmented both I_mAHP_ and I_sAHP_ by 81.1% and 64.4% (n = 6) respectively (Fig. 3B,C,D). Application of nimodipine (10 μM) inhibited the augmented I_mAHP_ by 60.0 ± 9.6% (298.0 ± 33.1 vs 129.7 ± 46.3 pA; *p* = 0.0013, paired two-tailed Student’s *t*-test) (n = 6) and I_sAHP_ by 75.1 ± 4.9% (120.1 ± 7.7 vs 31.2 ± 6.7 pA; *p* < 0.0001, paired two-tailed Student’s *t*-test) (n = 6) (Fig. 3C,D). The amplitude of both currents in the presence of nimodipine was not significantly different between control and 4R0N-tau-expressing cells (I_mAHP_, *p* = 0.8; I_sAHP_, *P* = 0.37, unpaired two-tailed Student’s *t*-test), indicating that augmentation of both current components by 4R0N-tau resulted from nimodipine-sensitive current. These data suggest that expression of 4R0N-tau enhances Ca^2+^ entry through L-type channels to increase the amplitude of the Ca^2+^-dependent currents that underlie the medium and slow AHPs.

### Augmentation of L-type channel current by 4R0N-tau

Hippocampal neurons express both Ca_V_1.2 and 1.3 isoforms of L-type Ca^2+^ channels^28^, which are pharmacologically indistinguishable under voltage-clamp conditions within a brain slice^29^. Therefore, we elected to determine the effect of different tau isoforms on expressed recombinant channel current.

**Figure 4:**
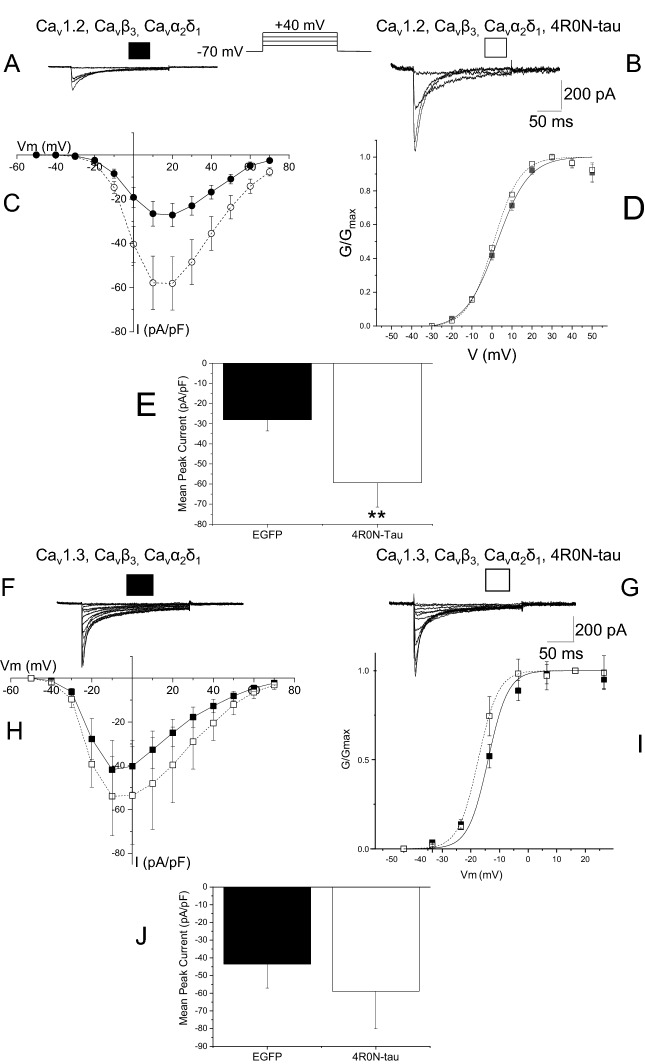
Co-expression of 4R0N-tau augments macroscopic Ca_V_1.2-mediated L-type Ca^2+^ current. Representative whole-cell Ca^2+^ currents from tsA-201 cells expressing Ca_V_1.2, Ca_V_β3, Ca_V_α2δ1 subunits in the absence (**A**) (●) (n = 19) and presence (**B**) (◯) of co-expressed 4R0N-tau (n = 19). Enhancement of macroscopic current was observed throughout the voltage range (**C**) and was not accompanied by a shift in activation (**D**) (V_50_ EGFP: 2.42 ± 0.98 mV, V_50_ 4R0N-tau: 2.17 ± 1.33 mV). (**E**) Co-expression of 4R0N-tau caused a significant increase in peak Ca_V_1.2-mediated L-type current density from − 28.0 ± 5.6 pA/pF to − 59.4 ± 12.1 pA/pF (**p > 0.02). Representative whole-cell macroscopic currents from tsA-201 cells expressing Ca_V_1.3, Ca_V_β3, Ca_V_α2δ1 subunits in the absence (**F**) (●) and presence (**G**) (◯) of co-expressed 4R0N-tau. As observed with Ca_V_1.2-mediated current, enhancement of Ca_V_1.3-mediated current was observed throughout the voltage range (**H**) without any change in voltage dependence of activation (**I**). (**J**) The enhancement of peak Ca_V_1.3-mediated L-type current density by co-expression of 4R0N-tau did not reach significance (*p* = 0.53).

Expression of the pore-forming subunit Ca_V_1.2 and auxiliary Ca_V_α2δ1 and Ca_V_β3 subunits in tSA-201 cells produced inward Ca^2+^-carried current that activated from − 30 mV, peaking around + 10 mV. Evoked current displayed prominent decay during the 250 ms depolarizing voltage step (Fig. 4). Co-expression of 4R0N-tau produced Ca_V_1.2-mediated currents of larger amplitude (Fig. 4B). Normalising current amplitude to cell capacitance showed that co-expression of functional Ca_V_1.2 channels with 4R0N-tau enhanced current throughout the voltage range (Fig. 4C). Cells expressing Ca_V_1.2, Ca_V_α2δ1 and Ca_V_β3 subunits produced whole-cell Ca^2+^ current that had a peak amplitude of − 28.0 ± 5.6 pA/pF at + 20 mV (n = 19). Co-expression of 4R0N-tau augmented peak Ca_V_1.2-mediated current, giving a peak current at + 20 mV of − 59.4 ± 12.1 pA/pF (n = 19) (*p* = 0.2) (Fig. 4E). Construction of peak current activation curves showed that augmentation of current by 4R0N-tau was not accompanied by a change in voltage dependence of activation (Fig. 4D) (control V_0.5_ 2.4 ± 1.0 mV, 4R0N-tau-expressing V_0.5_ 2.2 ± 1.3 mV).

Neuronal L-type calcium current is also mediated by channels containing the pore-forming subunit Ca_V_1.3. Co-expression of the Ca_V_1.3 subunit with auxiliary Ca_V_α2δ1 and Ca_V_β3 subunits produced a current that activated more negative (− 40 mV) and showed a greater degree of current decay during the depolarizing voltage step than seen with Ca_V_1.2/Ca_V_α2δ1/Ca_V_β3-mediated current^29,30^ (Fig. 4F). Currents peaked approximately 30 mV more negative (at − 10 mV) than that derived from expression of Ca_V_1.2/Ca_V_α2δ1/Ca_V_β3 subunits (Fig. 4H). Peak amplitude at − 10 mV was − 43.5 ± 13.6 pA/pF (n = 8) (Fig. 4J). Co-expression of 4R0N-tau augmented Ca_V_1.3/Ca_V_α2δ1/Ca_V_β3-mediated inward current (Fig. 4G,H,J), increasing current density to − 58.9 ± 21.2 pA/pF (n = 6) (Fig. 4J). Enhanced current was not accompanied by changes in voltage dependence of activation (Fig. 4I) (control V_0.5_ − 11.1 ± 1.6 mV, 4R0N-tau V_0.5_: − 22.3 ± 9.3 mV). It is clear that there was a trend to increase current amplitude upon co-expression of 4R0N-tau, but the effect did not reach significance (*p* = 0.53).

### Tau-mediated augmentation of L-type channel current is dependent on CaVβ

**Figure 5:**
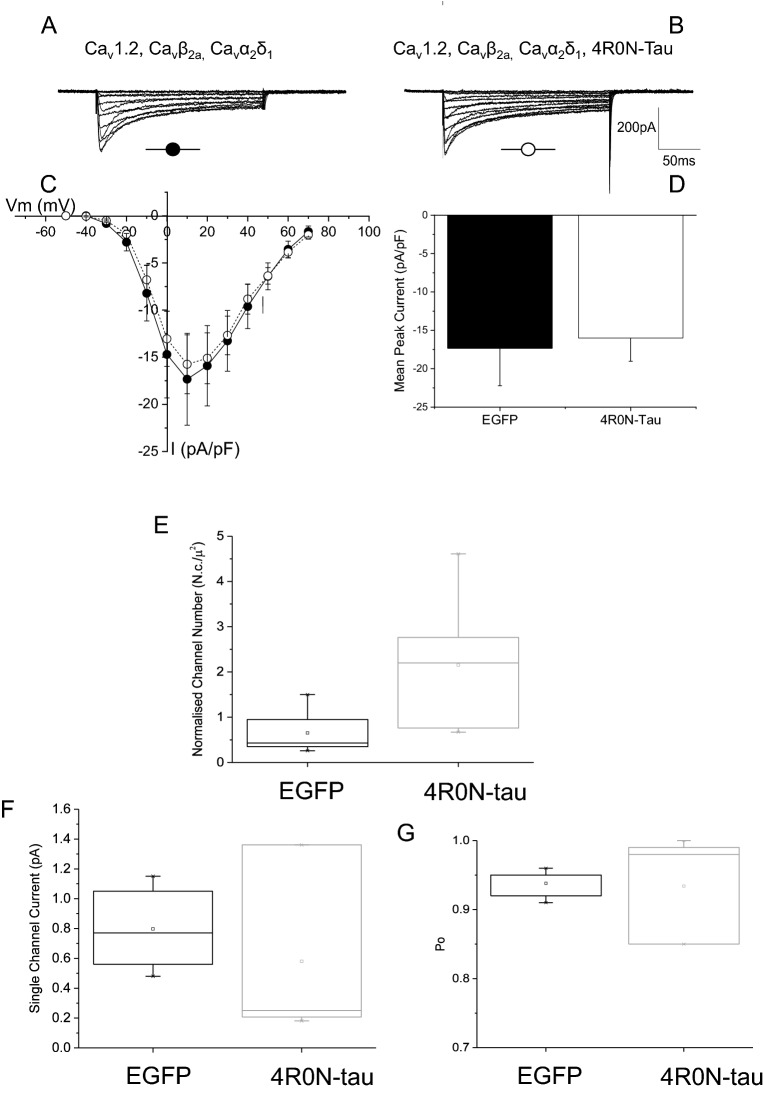
Effect of 4R0N-tau co-expression is dependent on the Ca_v_β subunit. (**A**–**D**) Co-expression of 4R0N-tau had no effect on the amplitude or voltage-dependence of activation of expressed Ca_v_1.2/Ca_v_α_2_δ_1_ channels when they are expressed with the Ca_v_β_2a_ subunit in tsA-201 cells. 4R0N-tau-mediated increase in Ca_V_1.2-mediated L-type Ca^2+^ current results from an increase in the number of functional channels. (**E**) Non-stationary noise analysis of Ba^2+^-carried Ca_v_1.2, Ca_v_β_3_, and Ca_v_α_2_δ_1_ L-type current showed that co-expression of 4R0N-tau (n = 7) increased channel density when compared with current from cells expressing EGFP alone (n = 6). In contrast, co-expression of 4R0N-tau had no effect on either single channel current amplitude (**F**) or channel open probability (Po) (**G**).

The most prevalent Ca_V_β subunits in hippocampal neurons are Ca_V_β2a and Ca_V_β3^31^. We tested the effects of 4R0N-tau expression on Ca_V_β2a-containing Ca_V_1.2 or 1.3 L-type channel current. Expression of Ca_V_1.2, Ca_V_α2δ1 and Ca_V_β2a subunits in tSA-201 produced inward Ca^2+^ current from − 30 mV (holding potential − 70 mV) (Fig. 5A). Co-expression of 4R0N-tau had no effect on the amplitude of evoked current (control peak current density − 17.3 ± 4.9 pA/pF, n = 5; 4R0N-tau-expressing − 16.0 ± 3.0 pA/pF) (*p* = 0.82) (Fig. 5B–D). The same lack of effect of co-expression of 4R0N-tau was observed on macroscopic current mediated by Ca_V_1.3 channels that contained the Ca_V_β2a subunit (Fig. S3). Inward current was evoked from cells transfected and expressing Ca_V_1.3, Ca_V_α2δ1 and Ca_V_β2a subunits from about − 40 mV and peaked around − 10 mV when Ca^2+^ was the charge carrier and slightly more negative when Ba^2+^ was used in place of Ca^2+^. Co-expression of these channel subunits with 4R0N-tau (Fig. S3) had little effect on current amplitude. These data indicate that augmentation of either Ca_V_1.2 or 1.3-mediated current by co-expression of 4R-tau is dependent on the identity of the auxiliary Ca_V_β subunit.

### Tau-mediated augmentation of L-type channel current results from an increase in functional channels

Augmentation of macroscopic current can involve increases in one or a combination of single channel current (i), open probability (Po) or the number of functional channels (N). It has been proposed that hippocampal L-type channel current increases in aged animals by an increase in the number of functional channels^18,19^. We used a non-stationary noise approach to estimate which of these parameters might underlie the increase in Ca_V_1.2-mediated L-type current by co-expression of 4R0N-tau. Cells expressing Ca_V_1.2, Ca_V_α2δ1 and Ca_V_β3 subunits were voltage-clamped at − 70 mV and subjected to a repeated depolarizing voltage step to the peak of the current–voltage relationship (+ 10 mV) (see Methods). This approach revealed that the co-expression of 4R0N-tau increased the amplitude of macroscopic current by increasing the number of functional channels (Fig. 5E). Analysis showed that the number of channels per cell significantly increased from 268 ± 76 in control EGFP-transfected cells (n = 7) to 1037 ± 215 in 4R0N-tau co-transfected cells (n = 7) (*p* = 0.03). Normalizing each cell estimate of channel number to the cell capacitance allowed us to estimate that channel density increased from 0.9 ± 0.2 channels/μm^2^ in control cells to 2.5 ± 0.6 channels/μm^2^ in cells co-expressing 4R0N-tau (Fig. 5E). Estimation of the amplitude of single channel current from this analysis showed that it did not increase, being − 0.80 ± 0.11 pA (n = 6) for control Ca_V_1.2/Ca_V_α2δ1/Ca_V_β3-mediated current and − 0.58 ± 0.20 pA (n = 7) in cells co-expressing 4R0N-tau (Fig. 5F) (*p* = 0.39). Finally, co-expression of 4R0N-tau had no significant effect (*p* = 0.91) on channel open probability (Po) of Ca_V_1.2/Ca_V_α2δ1/Ca_V_β3 channels (Fig. 5G). Cells transfected with Ca_V_1.2/Ca_V_α2δ1/Ca_V_β3 subunits alone gave a current that exhibited a channel Po of 0.94 ± 0.01 (n = 6), while co-expression with 4R0N-tau produced a Ca_V_1.2/Ca_V_α2δ1/Ca_V_β3-mediated current with a channel Po of 0.93 ± 0.03 (n = 7) (*p* = 0.91) (Fig. 5G).

### Tau isoform-specific augmentation of L-type channel current

**Figure 6:**
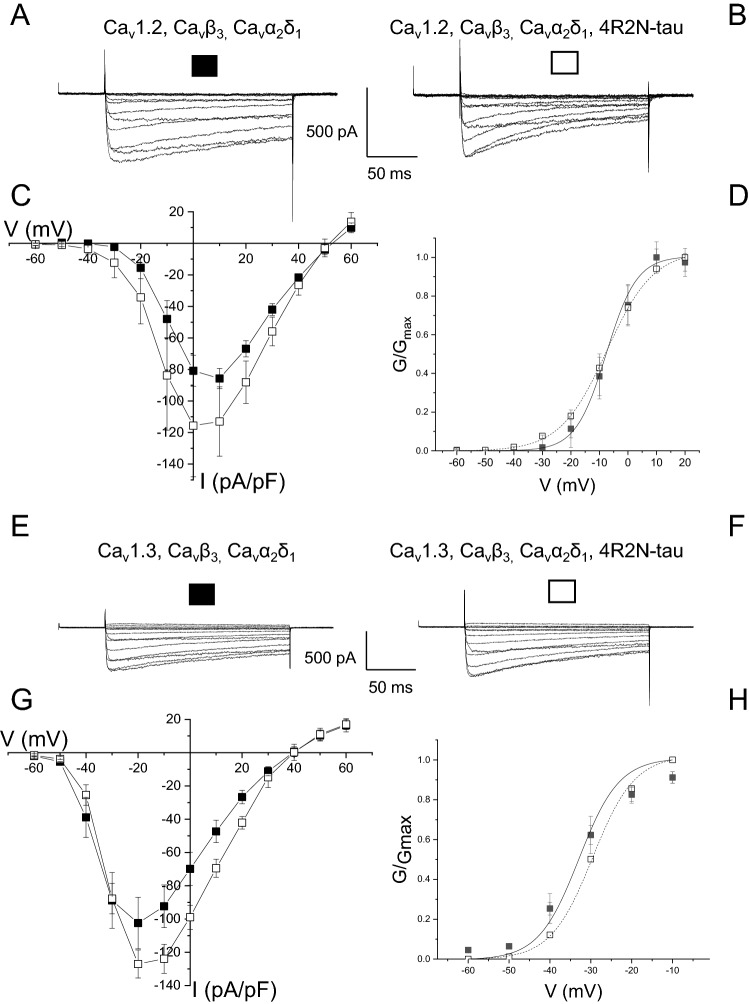
Both Ca_V_1.2- and CaV1.3-mediated L-type Ca^2+^ current is enhanced by co-expression of 4R2N-tau. (**A**) Representative macroscopic current traces from a tsA-201 cell expressing Ca_v_1.2, Ca_v_β_3_, and Ca_v_α_2_δ_1_ subunits evoked by step depolarizations from a holding potential of − 90 mV. (**B**) Augmented L-type current amplitude observed in cells co-expressing 4R2N-tau and Ca_v_1.2, Ca_v_β_3_, and Ca_v_α_2_δ_1_ subunits. (**C**) The amplitude of expressed Ca_v_1.2, Ca_v_β_3_, and Ca_v_α_2_δ_1_-mediated current was augmented throughout the voltage range when co-expressed with 4R2N-tau, with no shift in sensitivity to voltage (**D**). (**E**,**F**) Macroscopic Ca_v_1.3, Ca_v_β_3_, and Ca_v_α_2_δ_1_-mediated current evoked from a holding potential of − 90 mV, in the absence (**E**) and presence (**F**) of co-expressed 4R2N-tau. (**G**) Current–voltage relationship of Ca_v_1.3,Ca_v_β_3_,Ca_v_α_2_δ_1_-mediated current showing co-expression of 4R2N-tau augmented current with a small effect on voltage-dependence of activation. (**H**) Activation curve showing co-expression of 4R2N-tau caused a non-significant shift in voltage sensitivity, with V_0.5_ shifting from − 33.2 ± 2.0 mV to − 30.3 ± 1.1 mV (n = 8) (t = − 1.24, *p* = 0.23).

**Figure 7:**
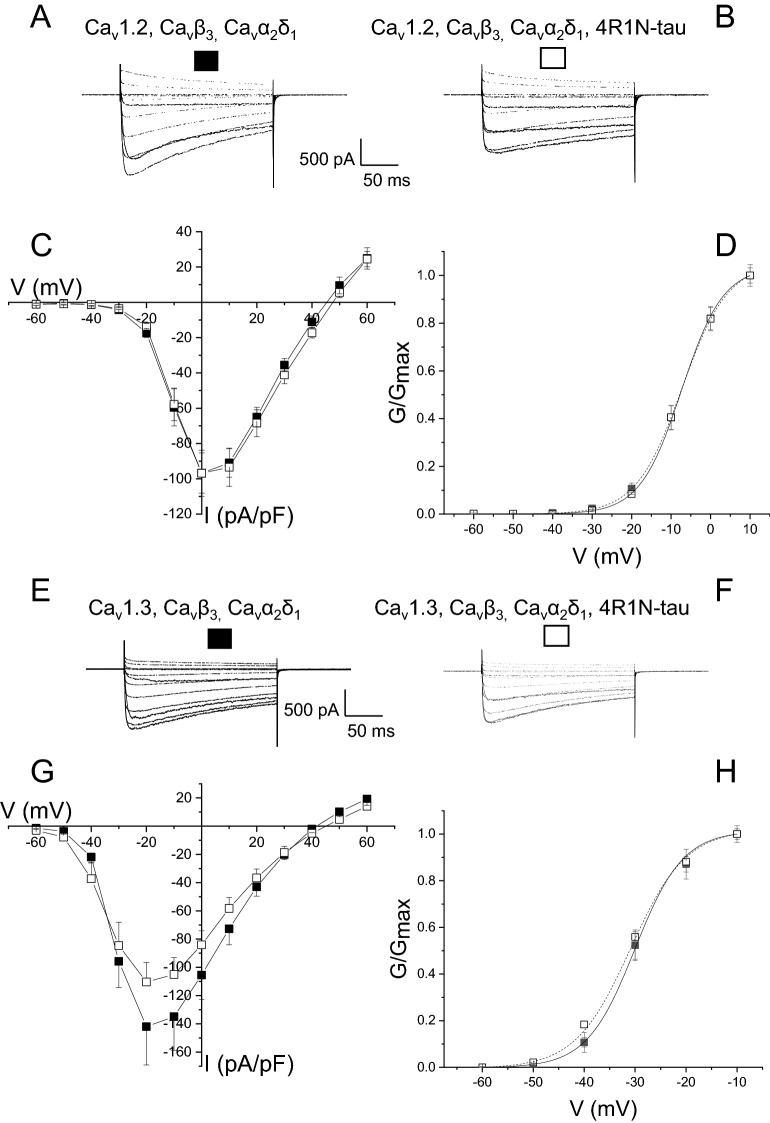
Co-expression of 4R1N-tau has no effect on L-type current. (**A,B**) Co-expression of 4R1N-tau in tsA-201 cells had no effect on macroscopic Ca_v_1.2, Ca_v_β_3_, and Ca_v_α_2_δ_1_-mediated current evoked from a holding potential of − 90 mV. Co-expression of the 4R1N-tau isoform had no effect on the current–voltage relationship (**C**) or activation curve (**D**) for the current. (**E**,**F**) In contrast, co-expression of 4R1N-tau caused a non-significant reduction in the amplitude of Ca_v_1.3, Ca_v_β_3_, and Ca_v_α_2_δ_1_-mediated current evoked from a holding potential of − 90 mV (**E**, EGFP-expressing cells − 146.2 ± 26.2 pA/pF (n = 8), ***F***, 4R1N-tau-expressing cells − 114.0 ± 14.6 pA/pF (n = 8) (t = 1.05, p = 0.30)). (**G**) Current–voltage relationship showing the small reduction in amplitude of Ca_v_1.3, Ca_v_β_3_, and Ca_v_α_2_δ_1_-mediated current was observed across the voltage range, with no effect on the voltage-dependence of activation (**H**).

Human tau has 6 isoforms, 3 containing 3R repeats, while the remainder contain 4R C-terminal repeats (3R and 4R). Each of the two groups contain isoforms that possess either no, one or two near N-terminal inserts (0N, 1N and 2N). It has become apparent that 4R isoforms of tau are implicated in dementia^24,25^ and it is important to test all 4R isoforms on L-type channel current. Co-expression of 4R2N-tau produced a trend to augment L-type currents in tSA-201 cells transfected with Ca_V_1.2, Ca_V_α2δ1 and Ca_V_β3 subunits (Fig. 6A–C). Peak current amplitude increased from − 92.5 ± 7.7 pA/pF in control (n = 9) to − 142.9 ± 28.5 pA/pF when co-expressed with 4R2N-tau (n = 10) (*p* = 0.06) (Fig. 6A–C). Current was evoked using Ba^2+^ as the charge carrier, showing that any effect of co-expression of tau is not dependent on the identity of the charge carrier. As was observed with the 4R0N-tau isoform, augmentation of macroscopic current by co-expression of 4R2N-tau was not accompanied by a shift in the voltage dependence of activation (control V_0.5_ − 4.8 ± 2.7 mV, 4R2N-tau-expressing V_0.5_ − 5.4 ± 4.0 mV) (Fig. 6D). A trend of an increase in the amplitude of current mediated by Ca_V_1.3, Ca_V_α2δ1 and Ca_V_β3 subunits by co-expression of 4R2N-tau was seen in a similar way as observed co-expression of 4R0N-tau (Fig. 6E–G), and not seen when the channel contained the Ca_V_β2a subunit (Fig. S4). Peak current amplitude was non-significantly increased from − 104.9 ± 15.5 pA/pF (n = 8) to − 128.6 ± 9.0 pA/pF (n = 8) (*p* = 0.2). Finally, co-expression of 4R1N-tau and either Ca_V_1.2 or Ca_V_1.3, with Ca_V_α2δ1 and Ca_V_β3 subunits, failed to have any effect on the amplitude of functional current (Fig. 7) (Peak current: Ca_V_1.2, Ca_V_α2δ1, Ca_V_β3 − 100.1 ± 10.8 pA/pF (n = 10); Ca_V_1.2, Ca_V_α2δ1, Ca_V_β3 + 4R1N-tau − 100.3 ± 12.7 pA/pF (n = 12) (p = 0.99); Ca_V_1.3, Ca_V_α2δ1, Ca_V_β3 − 146.2 ± 26.2 pA/pF (n = 8); Ca_V_1.3, Ca_V_α2δ1, Ca_V_β3 + 4R1N-tau − 114.0 ± 14.6 pA/pF (n = 9) (*p* = 0.3)). In addition, co-expression of 4R1N-tau had no effect on the voltage sensitivity of current activation (Fig. 7D,F) (Ca_V_1.2, Ca_V_α2δ1, Ca_V_β3 V_0.5_ − 14.9 ± 1.2 mV, Ca_V_1.2, Ca_V_α2δ1, Ca_V_β3 + 4R1N-tau V_0.5_ − 14.7 ± 1.1 mV; Ca_V_1.3, Ca_V_α2δ1, Ca_V_β3 V_0.5_ − 37.0 ± 5.2 mV, Ca_V_1.3, Ca_V_α2δ1, Ca_V_β3 + 4R1N-tau V_0.5_ − 39.0 ± 5.1 mV).

These data illustrate that augmentation of macroscopic L-type current is dependent on the 4R isoform of tau, with the greatest effect observed upon co-expression of 4R0N-tau. In addition, there is some selectivity of the effect of 4R isoforms of tau, with greater enhancement of current observed with channels containing the Ca_V_1.2 subunit.

### Tau isoform-specific association with Ca_V_β3 subunits

**Figure 8:**
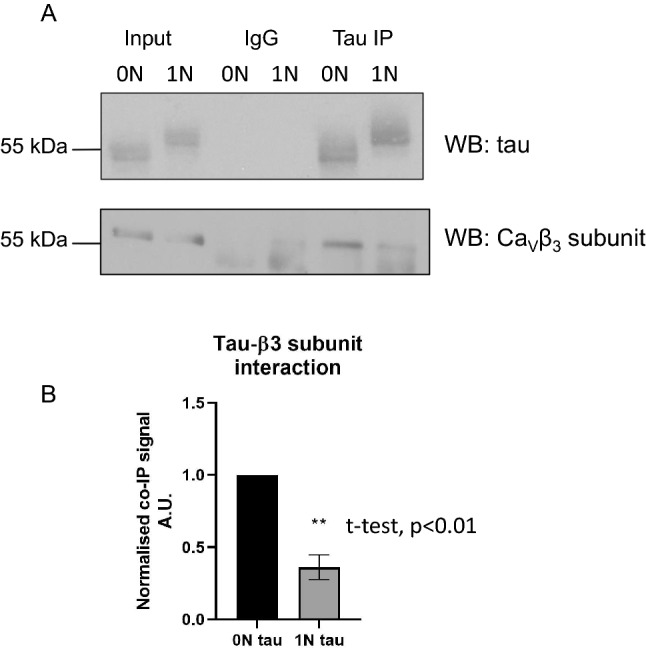
Interaction between tau variants and the Ca_V_β3 subunit through co-immunoprecipation. Lysates from tsA-201 cells expressing the Ca_V_β3 subunit together with either 4R0N- or 4R1N-tau were incubated for 1.5 h with either tau antibody or control IgG. Protein G sepharose beads were then added to precipitate the antibody:protein complex and bound proteins were analysed by Western blotting. (**A**) Western blotting with anti-tau antibody confirmed comparable variant expression and specific immunoprecipitation. Western blotting with anti-Ca_V_β3 showed that the 4R0N tau variant is more strongly associated with the Ca_V_β3 subunit when compared with 4R1N-tau. (**B**) The ratio of IP tau:Input tau for both 4R0N- and 4R1N-tau, and that for IP Ca_V_β3:Input Ca_V_β3 were calculated, followed by the calculation of the CaVβ3/tau ratio, with data presented normalised to the 4R0N-tau label intensity. Data quantification confirmed that there is a statistically significant difference between β3 association with the two tau variants (n = 3, t test, **p < 0.01).

Tau binds to microtubules to promote assembly and stabilization^32^. Tau has been also shown to directly interact with other proteins. For example, 4R0N-tau binds to Synaptogyrin-3 and thereby associates with presynaptic vesicles^33^, while 4R2N-tau interacts directly with heat shock protein (HSP) 90^34^ and 14-3-3 proteins^35^. We used a co-immunoprecipitation approach to determine whether the tau-isoform dependent augmentation of functional L-type channel current results from a direct interaction. The augmentation of current by co-expressed 4R0N-tau is dependent on the channel containing the Ca_V_β3 subunit, therefore cells were transiently transfected with plasmids encoding *either* 4R0N-tau *or* 4R1N-tau, and the Ca_V_β3 subunit. Cell lysates were incubated with control IgG or tau antibody for co-immunoprecipitation, followed by Western blot analysis with anti-tau or anti-Ca_V_β3 (Fig. 8, see Fig. S5 for uncropped blots). Incubation with control IgG did not result in immunoprecipitation of any expressed protein, whereas anti-tau effectively immunoprecipitated 4R0N- and 4R1N-tau. Ca_V_β3 showed a robust and specific interaction with 4R0N-tau, in contrast to a significantly weaker interaction with 4R1N-tau (Fig. 8A). Data normalised for Ca_V_β3 expression and tau immunoprecipitation showed a 64% reduction in the level of Ca_V_β3-bound 4R1N-tau compared to 4R0N-tau (n = 3, t-test, p < 0.01) (Fig. 8B). This greatly reduced association between 4R1N-tau and the Ca_V_β3 subunit is consistent with the lack of a significant augmentation of functional Ca_V_1.2, Ca_V_α2δ1, Ca_V_β3-mediated current (Fig. 7).

## Discussion

The entry of Ca^2+^ through L-type channels has been shown to activate the potassium channels that underlie generation of the Ca^2+^-dependent components of the medium and slow AHPs^11,27^. Expression of a 4R isoform of human tau augmented both AHP components, an effect resulting from an increase in the amount of Ca^2+^ entry through L-type channels (Figs. 1, 2, 3). This increase in Ca^2+^ entry (Fig. 4) resulted from an increase in the number of functional channels in the membrane (Fig. 5). The effect of expression of tau was not seen with 4R1N-tau, but was observed with both 4R0N and 4R2N isoforms (Figs. 4, 6, 7). L-type Ca^2+^ channels are multi-subunit complexes and require a beta subunit that regulates trafficking and biophysical properties of the functional channel. The effect of tau expression to augment macroscopic L-type channel current was only observed when channels possessed the Ca_V_β3 subunit (Fig. 4) and not when expressed channels contained the Ca_V_β2a subunit (Fig. 5). Finally, the identity of the α1 pore-forming subunit affected the magnitude of enhancement of macroscopic L-type current by expression of 4R0N and 4R2N isoforms of tau. L-type channels containing the Ca_V_α1.3 subunit were less sensitive to co-expressed 4R0N- or 4R2N-tau than channels containing the Ca_V_α1.2 subunit, despite both containing the Ca_V_β3 subunit (Figs. 4, 6).

Co-expression of channels and membrane associated proteins can affect macroscopic current. For example, sodium channel expression increases with co-expression of the microtubule-associated protein Map1b^36^. The auxiliary Ca_V_α2δ3 subunit associates with neurexin (NRX-1) to decrease Ca_V_2.2-mediated whole-cell current^37^. Co-expression of Rab interacting molecules (RIMs) 2α and 3γ augmented Ca_V_1.3/Ca_V_β2a/ Ca_V_α2δ1-mediated current by directly interacting with the C-terminus of the α1 pore-forming subunit^38^. Our data suggests that the physical association between 4R0N-tau and Ca_V_β3 results in a stabilization of functional Ca_V_1.2-containing channels in the membrane, leading to augmented Ca^2+^ entry. In addition, we observed a similar augmentation of evoked AHPs in hippocampal neurons expressing 4R0N(P301L)-tau (data not shown). This mutation within tau that is present in Hereditary Frontotemporal Dementia and Parkinson’s disease which is linked to chromosome 17 (FTDP-17) augments co-expressed L-type channel current^39^. Mice over-expressing this mutation (P301L) exhibit a larger AHP in dorsal entorhinal cortical neurons^40^. Hippocampal neurons isolated from older animals displayed greater protection by nimodipine from Ca^2+^-mediated excitotoxicity than neurons from younger animals^41^, consistent with a reported increase in L-type channel current in hippocampal neurons from older animals^18^. The increase in L-type current is accompanied by a decrease in overall expression of both Ca_V_1.2 and 1.3 subunits, which is countered by more channels in the membrane as measured by surface biotinylation^19^. It is proposed that this increase in L-type channel current underlies the loss of hippocampal neurons as a common neuropathological feature in old age^42^. The expression of tau increases in age^43^ and we propose that the demonstrated physical interaction with tau stabilizes L-type channels in the membrane to augment Ca^2+^ entry. We consider that this enhanced entry of Ca^2+^ augments AHPs in hippocampal neurons, inhibiting action potential firing and possibly producing cognitive deficits^15^.

The presence of amyloid β42 and tau proteins in cerebrospinal fluid (CSF) are core biomarkers for the diagnosis of AD^44^. The levels of tau are elevated in the CSF of sufferers of AD and there is evidence that a greater proportion of 4R-tau isoforms are present^45^. Normal human adult hippocampus contains approximately equal amounts of 3R-tau and 4R-tau isoforms^46^, with a shift in the ratio being proposed to result in early cognitive impairments^47,48^. For example, it has been suggested that an excess of 4R-tau relative to 3R-tau isoform produces neurodegeneration in *Drosophila*^49^. Abundance of 4R isoforms of soluble tau have been reported in patients with Alzheimer’s disease, progressive supranuclear palsy and Pick’s disease^50^. It is plausible that the increased number of L-type Ca^2+^ channels in hippocampus of humans diagnosed with AD^51^ results from association of the channel with elevated expression of 4R-tau isoforms.

Dysfunction of how the levels of Ca^2+^ are regulated within a neuron has been implicated in AD and tauopathies^52^. For example, the intracellular Ca^2+^ level within cortical neurons of a mouse transgenic model of AD (3xTg-AD) was twice that observed in control cortical neurons, and this dysfunction was caused by both changes in Ca^2+^ entry and release from internal stores^53^. Our data suggest a mechanism for the regulation of cellular Ca^2+^ levels by over-expression of a 4R-tau isoform. We propose that the demonstrated interaction between the 4R-tau isoform and Ca_V_β3 subunit results in an increase in the number of functional L-type Ca^2+^ channels in the membrane.

It is certain that cognitive impairment occurs prior to diagnosis of tauopathies, but it is not known whether this results from cell loss in defined brain regions. The mild cognitive impairment observed in the early stages of tauopathies may result from the increased surface expression of functional L-type Ca^2+^ channels. This increase has been shown to produce an enhanced AHP following a train of action potentials that would inhibit subsequent firing. This enhancement of the AHP mirrors that seen in aged animals, where suppression of the AHP by a L-type channel blocker aids learning^14^. It is suggested that a similar enhancement produced by wild-type tau expression would produce mild cognitive impairment. Finally, AD and tauopathies are characterized by cell loss, a process that has been proposed to involve dysregulation of intracellular Ca^2+^ levels. The increased level of intracellular Ca^2+^ within cortical neurons of a mouse transgenic model of AD (3xTg-AD) was largely reduced by inhibition of L-type Ca^2+^ channels^53^. These data suggest that stabilization of L-type Ca^2+^ channels in the plasma membrane by association with tau could result in both early cognitive impairment and contribute to cell loss in the later phase of AD or tauopathies.

## Methods

### Organotypic hippocampal slice cultures

Organotypic hippocampal slice cultures were prepared from 18 to 20-day-old male Wistar rats as described previously^27^. Rats were killed by cervical dislocation in accordance with Schedule 1 of the UK home office guidelines. All procedures were carried out in accordance with the UK Animal (Scientific Procedures) Act, 1986, and EU Directive 2010/63/EU. All experimental procedures were reviewed by the University of Bristol Ethical Review Group (reference: UB/12/006) and were in accordance with ARRIVE guidelines. Brains were removed and horizontal brain slices (300 µM) were cut in ice-cold (~ 4 °C) sucrose-based cutting solution containing (in mM): sucrose, 189; D-glucose, 10; NaHCO_3_, 26; KCl, 3; MgSO_4_.7H_2_O, 5; CaCl_2_, 0.1; NaH_2_PO_4_, 1.25; saturated with 95% O_2_ and 5% CO_2_ using a VT1000 S vibrating blade microtome (Leica Microsystems Ltd, Milton Keynes, UK). After sectioning, hippocampal slices were transferred to a storage chamber filled with artificial cerebrospinal fluid (aCSF) containing (in mM): NaCl, 124; KCl, 3; NaHCO_3_, 24; NaH_2_PO_4_.H_2_O, 1.25; MgSO_4_.7H_2_O, 1; D-glucose, 10 (saturated with 95%O_2_/5% CO_2_)_._ Slices were washed three times under aseptic conditions with culture media containing Minimum Essential Medium (Gibco) supplemented with (in mM): NaHCO_3_, 5; HEPES, 75; glutamine, 0.437; CaCl_2_, 0.625; MgSO_4_.7H_2_O, 1.25; ascorbic acid, 0.425; D-glucose, 32; 12.5% heat-inactivated horse serum, 49.4% MEM, 1 mg/ml insulin, and 50 units/ml penicillin with 50 µg/ml streptomycin (pH 7.28 with NaOH) (320 mOsm). Slices were washed a further three times in culture media without supplemented penicillin–streptomycin. Slices were maintained at 37 °C (5% CO_2_) for 3 days on Millipore (Bedford, MA) membrane culture inserts (Millicell CM; pore size, 0.4 µM) in six-well culture plates with 1 ml culture media.

### Preparation and transduction of modified Sindbis virus encoding 4R0N-tau in organotypic slice cultures

Rat neurons contain all 6 isoforms of tau that are expressed in human neurons, with a similar balance between 3 and 4R isoforms to human^54^. Rat provides a background of normal tau expression to permit determination of the effect of over-expression of one 4R-tau isoform.

Human 4R0N-tau cDNA was subcloned into pIRES-EGFP2. Human 4R0N-tau-IRES-EGFP2 was subsequently excised and subcloned into the linearized pSinRep5(nsP2S^726^) viral expression vector (which was generously donated by Prof. Jeremy Henley, University of Bristol, UK) and confirmed by DNA sequencing (Source Bioscience, Oxford). Attenuated Sindbis virus was prepared and used as previously reported (Kim et al., 2004; Martin et al., 2007). In brief, cRNA was synthesised with mMESSAGE mMACHINE SP6 (Ambion, Life Technologies) kit after vector linearizaton of pSinRep5(nsP2S^726^)-4R0N-tau-IRES-EGFP2 and pSinRep5(nsP2S^726^)-IRES-EGFP2. Following in vitro transcription, pSinRep5(nsP2S^726^)-4R0N-tau-IRES-EGFP2 or IRES-EGFP2 were mixed with the cRNA of the defective helper plasmid (pDH-BB) before the recombinant RNA was electroporated (Bio-Rad) into BHK-21 (C-13) cells. 48-h after electroporation, the culture medium containing the pseudovirions was centrifuged after harvesting to remove cell debris, and subsequently stored at − 80 °C in small aliquots.

Organotypic slices were infected using the droplet method on the second day in culture. Slices were incubated with the virus for 24 h. Expression of human 4R0N-tau isoform was confirmed in rat CA1 pyramidal neurons within organotypic hippocampal slices by immunohistochemistry, using a primary monoclonal anti-tau (Sigma Aldrich, Tau5) specific for the human 3R0N- and 4R0N-tau isoforms. Nonspecific binding was blocked by incubating slices with 5% fetal calf serum, 1% bovine serum albumin and 0.1% triton X-100 in PBS overnight at 4 °C. Slices were incubated with anti-tau for 2 days at 4 °C. Slices were then washed in phosphate buffered saline (PBS) before incubation with Alexa Flour 647 goat anti-mouse IgG_1_ far red (1:500, Life Technologies) in blocking solution for 3 days at 4 °C. Slices were washed in PBS and mounted in Hydromount mounting medium and labelling was visualised with a Leica AOBS SP2 confocal laser scanning microscope (Leica, Solms, Germany), using a 63x, NA oil immersion lens. Images were processed using Volocity Softare (PerkinElmer, Waltham, MA) (Fig. S1).

### Electrophysiology

Slices were continuously perfused (2–3 ml/min) with aCSF supplemented with NBQX (2,3-dihydroxy-6-nitro-7-sulfamoyl-benzo[f]quinoxaline-2,3-dione) (10 µM) to inhibit spontaneous AMPA receptor-mediated excitatory post-synaptic currents. Experiments performed in Ca^2+^-free aCSF contained a four-fold increase in Mg^2+^ concentration. The aCSF temperature was maintained at ~ 33 °C. Whole-cell current-clamp recordings were made from visually identified pyramidal neurons from the CA1 region of the hippocampus using an infrared-light emitting diode mounted on an Axioskop2 microscope (Carl Zeiss), with fire-polished pipettes manufactured from borosilicate glass (1.5 mm O.D., 0.86 mm I.D.) containing (in mM): KMeSO_4_, 125; KCl, 10; NaCl, 10; HEPES, 20; MgATP, 2; NaGTP, 0.3; EGTA, 0.2 (pH 7.3, 280–285 mOsm (pipette resistance 3–5 MΩ). A liquid junction potential error was experimentally measured (+ 13 mV) and was compensated for during current-clamp recordings. The membrane voltage for all recordings was recorded in the bridge-balance mode of the MultiClamp 700A amplifier (Molecular Devices, CA, USA). The membrane voltage was filtered at 1.2 kHz (eight-pole low-pass Bessel filter) and sampled at 5 kHz using Pulse (HEKA Electronics) and stored on a personal computer. Whole-cell voltage-clamp recordings were performed using an Axopatch 200B amplifier (Molecular Devices, Union City, Ca, USA). Signals were filtered at 2 kHz (8-pole low-pass Bessel filter, Frequency Devices, CT, USA) and digitised at 10 kHz. Whole-cell capacitance and series resistance were not compensated. Series resistance was continually monitored by measurement of peak current in response to a − 5 mV (10 ms) voltage step at the beginning of each sweep. Only data whereby the series resistance did not change by more than 25% over the duration of an experiment was included in subsequent analysis. I_AHP_ was observed following a depolarising step to + 10 mV (100 ms duration) from a holding voltage of − 50 mV.

### Data analysis

AHPs were elicited by evoking a train (15) of APs by brief (2 ms) 2 nA somatic current injections delivered at 50 Hz. Any cell that did not fire the correct number of APs was discarded. Analysis of the medium and slow AHPs was carried out using custom-written MatLab scripts (The MathWorks Company). The medium AHP was measured as the peak negative membrane deflection between 0 to 100 ms after the cessation of the last AP fired. The slow AHP was measured 1 s after the last AP was fired. The overlapping kinetic profiles of the medium and slow AHPs is minimized by measuring the medium AHP and slow AHP at these time points^27^. All recordings used cells with a stable resting membrane potential more negative than − 60 mV.

For voltage clamp recordings, data was analysed using Pulsefit (HEKA, Lambrecht/Pfalz, Germany). I_mAHP_ was measured from zero to peak current, while I_sAHP_ was measured 1 s after the end of the depolarizing voltage step.

### Drugs

All drugs were bath applied. NBQX was prepared as a stock solution in dimethylsulfoxide (DMSO) and diluted in aCSF when required. All salts were purchased from Sigma-Aldrich except HEPES, which was obtained from Merck Serono (Fletham, UK).

### TsA-201 cell electrophysiology

The tsA201 cell line was maintained as described previously^27^. Cells were transiently transfected using Lipofectamine LTX with plasmids encoding Ca_V_1.2 (GenBank™ accession number AF394940) or Ca_V_1.3 (GenBank™ accession number D38101), with either Ca_V_β2a (GenBank™ accession number M80545) or Ca_V_β3 (GenBank™ accession number M88751) and Ca_V_α2δ1 (GenBank™ accession number AF286488), with eGFP expression used as a marker for transfection. Using fluorescent proteins as a marker for ion channel expression is used widely and enables targeted recording without affecting channel function^55,56^. Cells were transfected while seeded in 25 cm^2^ flasks, using 1.2–2 μg of each channel subunit plasmid and 0.4 μg of plasmid encoding eGFP in 8 mls of DMEM. Experiments to resolve the effects of co-expression of a 4R-tau isoform were accomplished by using 2 μg of construct in addition to those listed above. Cells were seeded into 35 mm culture dishes 24 h after transfection and cells expressing enhanced green fluorescent protein were used for electrophysiology 24 h later.

Expressing cells were bathed in an external solution of composition (mM): TEACl, 140; HEPES(Na), 10; CaCl_2_, 10; MgCl_2_, 1 and D-glucose, 10 (pH 7.4, 320 mOsm) and whole-cell voltage-clamped using electrodes manufactured from KG-33 borosilicate glass containing (mM): CsCl, 120; TEACl, 20;﻿ HEPES(Na), 10;﻿ EGTA, 5;﻿ Na_2_ATP, 1.5 and MgCl_2_, 1.5 (pH 7.4, 280 mOsm). Electrode resistances were 2–5 MΩ. Membrane current was recorded using an Axopatch 200A and filtered at a cut-off frequency of 1 kHz and sampled at 10 kHz using Pulse (HEKA). Currents were evoked by step depolarizations from a holding potential of − 80 mV, with 70–90% series resistance compensation used throughout. Evoked currents were leak subtracted using a P/4 protocol from a holding potential of − 90 mV. Non-stationary noise experiments were conducted using Ba^2+^ as the charge carrier, where the external solution had an identical composition but where 10 mM BaCl_2_ was used in place of CaCl_2_. A single pulse protocol of a 200 ms step depolarization to + 10 mV (peak of the current–voltage relationship) imposed every 5 s and repeated for a minimum of 100 sweeps.

### Co-immunoprecipitation

TsA201 cells were grown in 6 cm dishes until 80% confluency was achieved. Cultures were transfected using polyethylenimine (PEI) (Sigma Aldrich) with a ratio of 3:1 PEI:DNA, using 4.5 μg plasmid DNA encoding either 4R0N- or 4R1N-tau (both in pcDNA3.1) together with 4.5 μg plasmid DNA encoding Ca_V_β3 (in pcDNA3.1). Cells were lysed 36–48 h after transfection in 1 ml co-IP buffer (150 mM NaCl, 20 mM HEPES, 0.5% triton X-100, pH 7.4) before a 15 min incubation on ice that was followed by 30 min centrifugation at 16,000 g at 4 °C. Samples were incubated for 20 min with 10 μl Protein G sepharose beads (GE Healthcare) for pre-clearing. For the co-IP, half of the sample was incubated with 4 μg mouse IgG (Sigma) and the other half with 4 μg mouse anti-tau (Sigma, T9450) for 1.5 h on a spinning wheel 4 °C. Protein G beads were then added (30 μl) before the samples were returned to the wheel for an additional hour. Samples were then washed with co-IP buffer four times before 60 μl Laemmli buffer (65 mM Tris, 25% glycerol, 2% SDS, 0.01% bromophenol blue, 5% β-mercaptoethanol) was added to each condition. 12% polyacrylamide gels were used to separate proteins based on molecular weight by SDS-PAGE using the BioRad electrophoresis system. Gels were run for 1.5 h at a constant voltage of 150 V in running buffer (25 mM Tris, 250 mM glycine, 0.1% SDS). Wet transfer onto PVDF membranes was performed at 400 mA for 1 h in transfer buffer (50 mM Tris, 40 mM glycine, 20% methanol). Membranes were blocked by incubation with 5% milk (Own brand powdered milk, Co-op) in PBS-T (PBS + 0.1% Tween) for one hour at room temperature while shaken. Primary rabbit antibodies were diluted 1/1000 (anti-tau Origene, TA325666; anti-Ca_V_β3 subunit Biorbyt, orb108858) in 5% milk PBS-T overnight at 4 °C. Anti-rabbit HRP-linked secondary antibody (1/10,000, GE Healthcare) was added after three 10 min PBS-T washes and left for 45 min at room temperature. Finally, membranes were subjected to 3 × 10 min washes with PBS-T, followed by a 1 min incubation with either the Classico, Crescendo (Millipore) or the Femto (Thermo Scientific) ECL substrates (depending on the intensity of the signal). The signal was detected by exposing and developing X-ray films which were then scanned in grayscale before using ImageJ for quantification. The co-IP data were normalised to the corresponding input signal values before a ratio of Ca_V_β3 bound to tau was calculated for the two tau variants. Statistical analysis was performed using GraphPad Prism.

### Data analysis

Statistical analysis of current clamp data was performed using SPSS (v21, IBM) and representative traces were drawn using Origin 9 (Microcal Software). All data is presented as mean ± SEM. Paired two-tailed Student’s *t*-tests were used to compare the means between control and 4R-tau-expressing groups. A repeated measures ANOVA was used to compare AHP amplitudes. All voltage clamp data analysed using Pulsefit (HEKA). Non-stationary noise analysis was performed using WinWCP V5.1.5 software. All statistical tests for significance were conducted using a non-paired Students t-test. Mean current values were calculated from individual cells and presented as ± standard error of the mean (SEM).

## Supplementary Information

Supplementary Figures.

## Data Availability

All materials, data and associated protocols will be made available to readers upon reasonable request to Prof. N.V. Marrion (N.V.Marrion@bristol.ac.uk), without undue qualifications.
