# Supplementary material for: Tau isoform-specific enhancement of L-type calcium current and augmentation of afterhyperpolarization in rat hippocampal neurons
Source: Sci Rep. 2022 Sep 8;12:15231. doi: 10.1038/s41598-022-18648-0 (PMC9458744; doi:10.1038/s41598-022-18648-0)
Supplement: Supplementary file 1 — Supplementary Figures. [file 41598_2022_18648_MOESM1_ESM.pptx]

## Slide 1
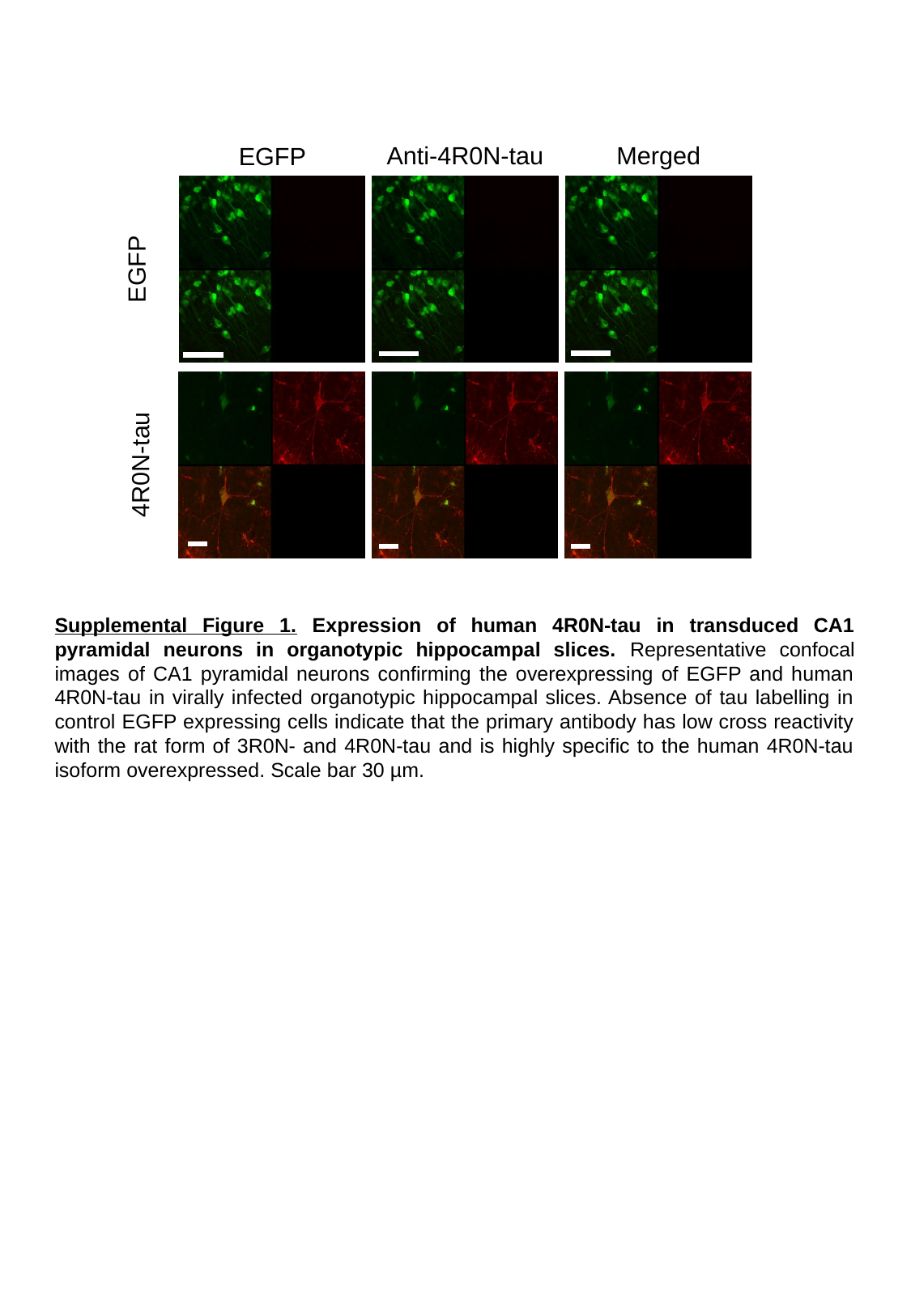

Anti-4R0N-tau
Merged
EGFP
EGFP
4R0N-tau
Supplemental Figure 1. Expression of human 4R0N-tau in transduced CA1 pyramidal neurons in organotypic hippocampal slices. Representative confocal images of CA1 pyramidal neurons confirming the overexpressing of EGFP and human 4R0N-tau in virally infected organotypic hippocampal slices. Absence of tau labelling in control EGFP expressing cells indicate that the primary antibody has low cross reactivity with the rat form of 3R0N- and 4R0N-tau and is highly specific to the human 4R0N-tau isoform overexpressed. Scale bar 30 µm.

## Slide 2
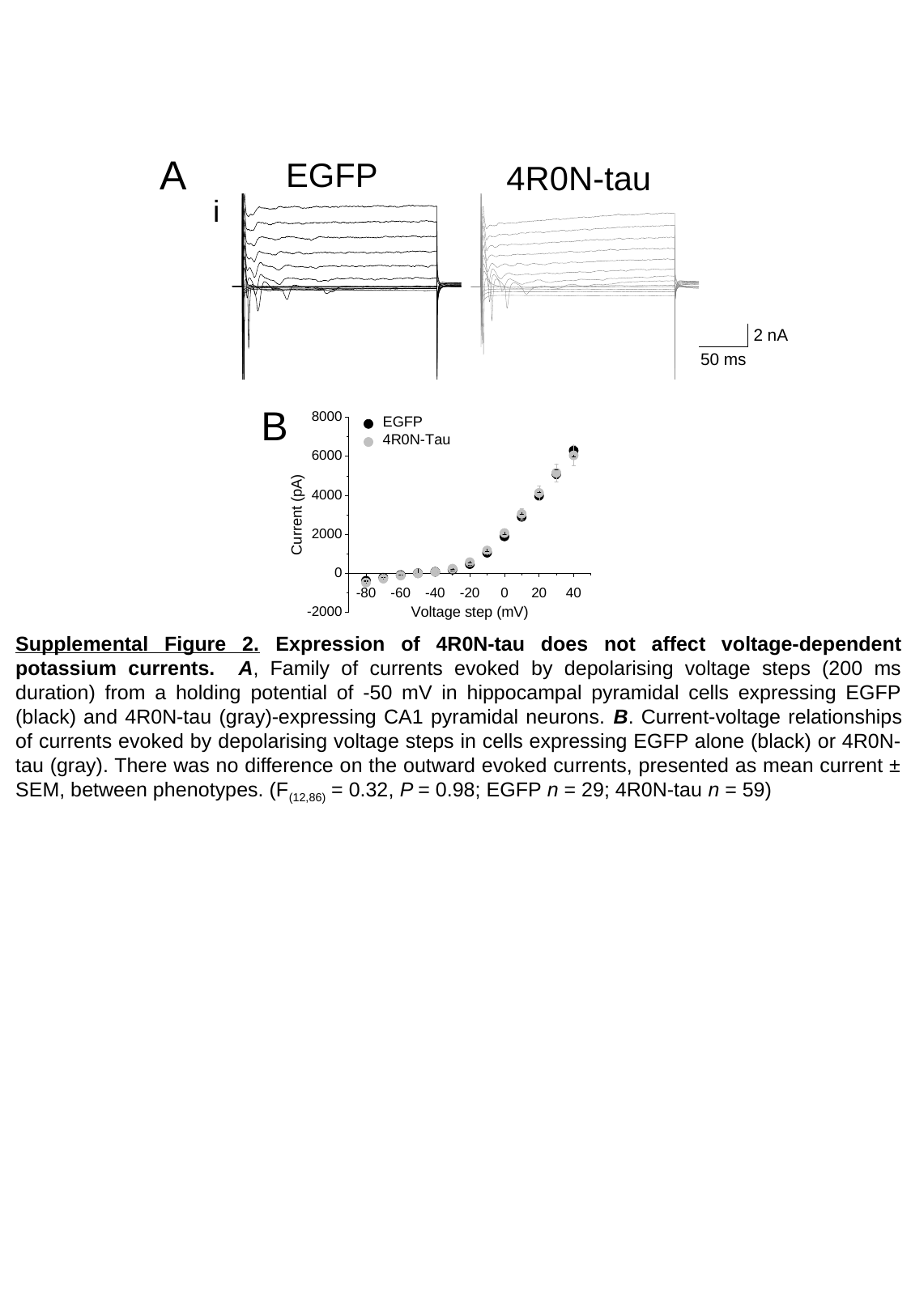

A
EGFP
4R0N-tau
2 nA
50 ms
i
B
Supplemental Figure 2. Expression of 4R0N-tau does not affect voltage-dependent potassium currents. A, Family of currents evoked by depolarising voltage steps (200 ms duration) from a holding potential of -50 mV in hippocampal pyramidal cells expressing EGFP (black) and 4R0N-tau (gray)-expressing CA1 pyramidal neurons. B. Current-voltage relationships of currents evoked by depolarising voltage steps in cells expressing EGFP alone (black) or 4R0N-tau (gray). There was no difference on the outward evoked currents, presented as mean current ± SEM, between phenotypes. (F(12,86) = 0.32, P = 0.98; EGFP n = 29; 4R0N-tau n = 59)

## Slide 3
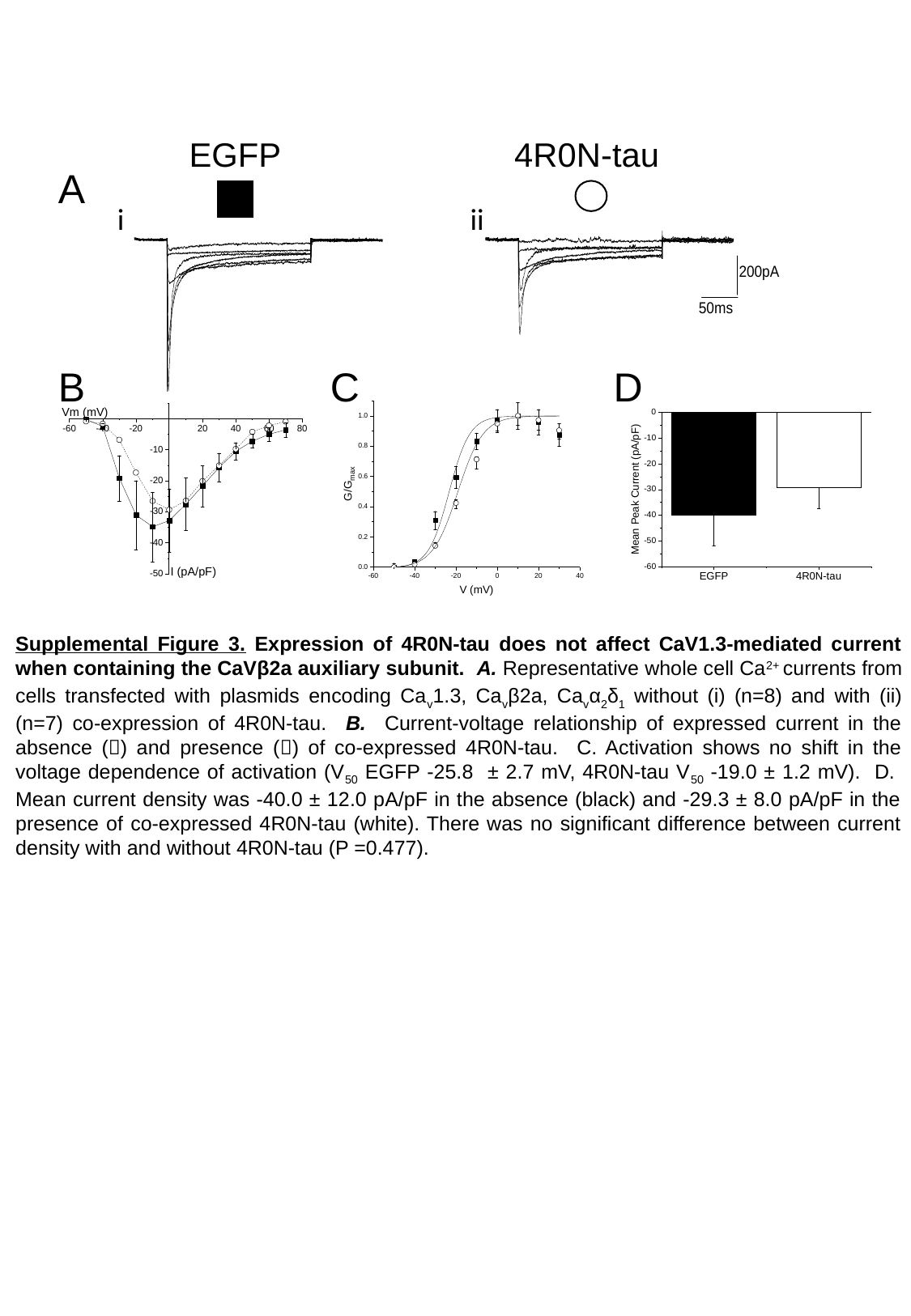

EGFP
4R0N-tau
A
i
ii
B
C
D
Supplemental Figure 3. Expression of 4R0N-tau does not affect CaV1.3-mediated current when containing the CaVβ2a auxiliary subunit. A. Representative whole cell Ca2+ currents from cells transfected with plasmids encoding Cav1.3, Cavβ2a, Cavα2δ1 without (i) (n=8) and with (ii) (n=7) co-expression of 4R0N-tau. B. Current-voltage relationship of expressed current in the absence () and presence () of co-expressed 4R0N-tau. C. Activation shows no shift in the voltage dependence of activation (V50 EGFP -25.8 ± 2.7 mV, 4R0N-tau V50 -19.0 ± 1.2 mV). D. Mean current density was -40.0 ± 12.0 pA/pF in the absence (black) and -29.3 ± 8.0 pA/pF in the presence of co-expressed 4R0N-tau (white). There was no significant difference between current density with and without 4R0N-tau (P =0.477).

## Slide 4
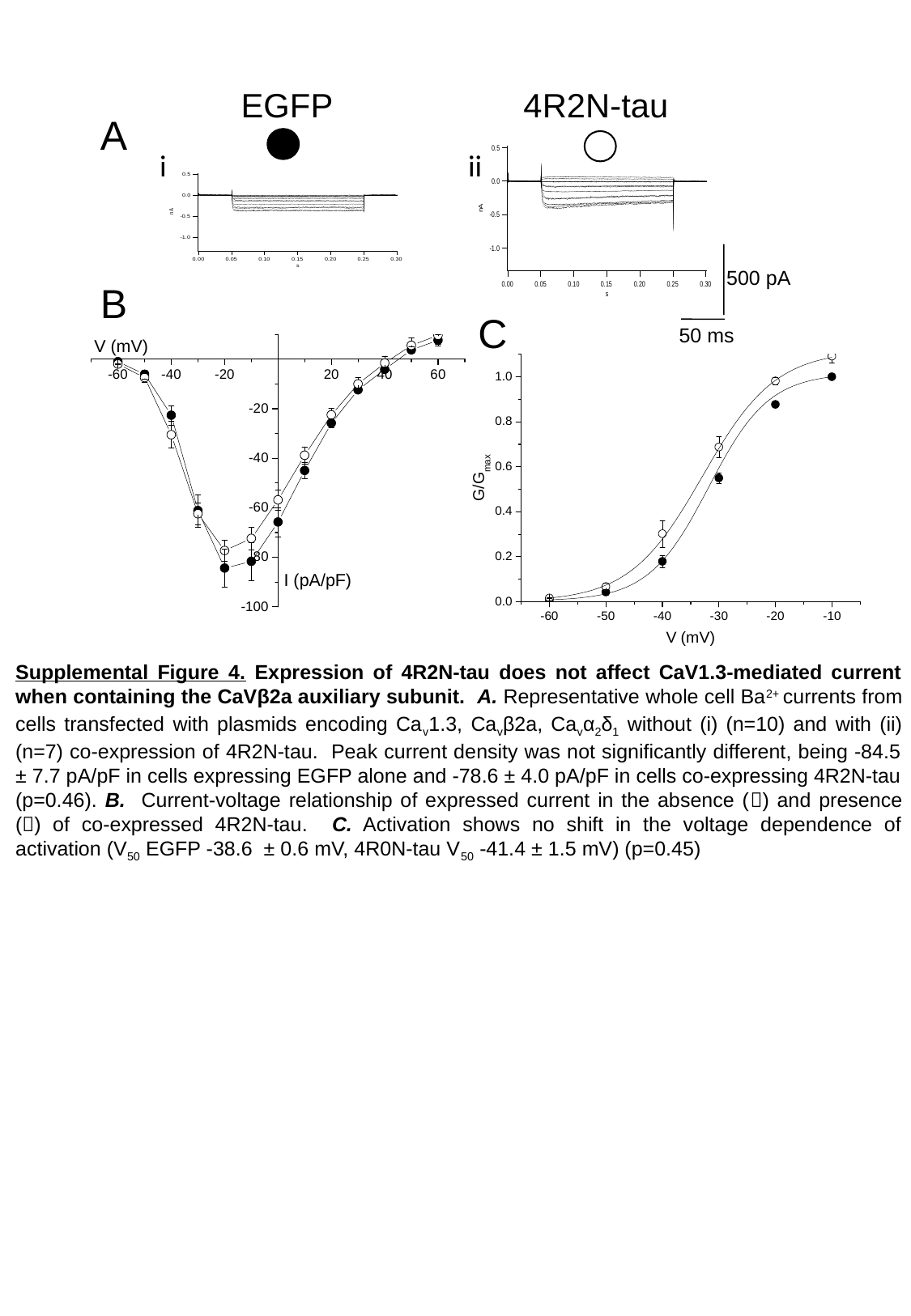

4R2N-tau
EGFP
A
i
ii
500 pA
50 ms
B
C
Supplemental Figure 4. Expression of 4R2N-tau does not affect CaV1.3-mediated current when containing the CaVβ2a auxiliary subunit. A. Representative whole cell Ba2+ currents from cells transfected with plasmids encoding Cav1.3, Cavβ2a, Cavα2δ1 without (i) (n=10) and with (ii) (n=7) co-expression of 4R2N-tau. Peak current density was not significantly different, being -84.5 ± 7.7 pA/pF in cells expressing EGFP alone and -78.6 ± 4.0 pA/pF in cells co-expressing 4R2N-tau (p=0.46). B. Current-voltage relationship of expressed current in the absence () and presence () of co-expressed 4R2N-tau. C. Activation shows no shift in the voltage dependence of activation (V50 EGFP -38.6 ± 0.6 mV, 4R0N-tau V50 -41.4 ± 1.5 mV) (p=0.45)

## Slide 5
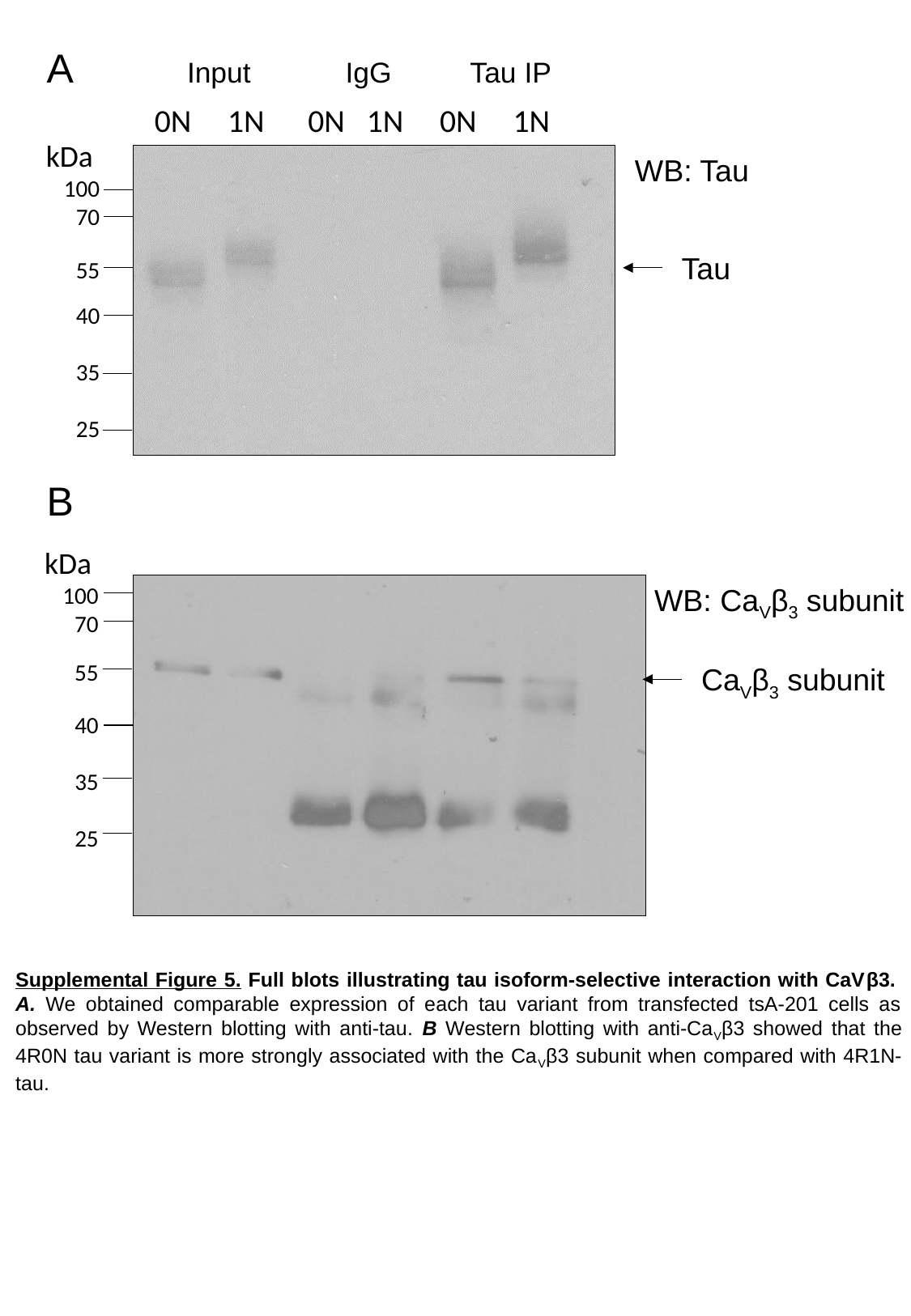

A
 Input IgG Tau IP
 0N 1N 0N 1N 0N 1N
kDa
100
70
55
WB: Tau
Tau
40
35
25
B
kDa
100
70
55
WB: CaVβ3 subunit
CaVβ3 subunit
40
35
25
Supplemental Figure 5. Full blots illustrating tau isoform-selective interaction with CaVβ3. A. We obtained comparable expression of each tau variant from transfected tsA-201 cells as observed by Western blotting with anti-tau. B Western blotting with anti-CaVβ3 showed that the 4R0N tau variant is more strongly associated with the CaVβ3 subunit when compared with 4R1N-tau.
